# Effects of Metamifop on Defense Systems in *Monopterus albus*

**DOI:** 10.3390/toxics11100811

**Published:** 2023-09-25

**Authors:** Tianyu Guan, Yi Zhang, Qianqian Zhu, Long Wang, Jianbin Feng, Hui Wang, Jiale Li

**Affiliations:** 1 Jiangsu Collaborative Innovation Center of Regional Modern Agriculture & Environmental Protection, Huaiyin Normal University, Huai’an 223300, China; guanty980716@163.com (T.G.);; 2 Key Laboratory of Freshwater Aquatic Genetic Resources, Ministry of Agriculture, Shanghai Ocean University, Shanghai 201306, China; 3 School of Oceanography, Ningbo University, Ningbo 315211, China; 4 National Demonstration Center for Experimental Fisheries Science Education, Shanghai Ocean University, Shanghai 201306, China

**Keywords:** herbicides, oxidative stress, antioxidation, immunity, inflammation, apoptosis

## Abstract

The effects of herbicides on non-target organisms in paddy fields have become a popular research topic. As a widely used herbicide, it is necessary to explore the potential toxicity of metamifop in non-target organisms, especially aquatic animals, in co-culture mode. In the present study, we evaluated the effects of metamifop (0, 0.2, 0.4, 0.6, and 0.8 mg/L) on the defense system (antioxidation, immunity, and apoptosis) in *Monopterus albus*. Reactive oxygen species (ROS) production, malondialdehyde (MDA) content, and protein carbonylation (PCO) increased significantly (*p* < 0.05) with the increasing metamifop concentration, resulting in oxidative damage. In the antioxidant system, superoxide dismutase (SOD) and catalase (CAT) activities increased significantly (*p* < 0.05) in the 0.2 mg/L treatment group compared with the control group, and decreased in 0.4, 0.6, and 0.8 mg/L treatment groups. Glutathione peroxidase (GPX) activity decreased significantly (*p* < 0.05) with the increasing metamifop concentration. In the immune system, white cell number (WCN) increased significantly (*p* < 0.05) in 0.2 mg/L treatment group, and then decreased with the increase in metamifop concentration. Compared with control group, acid phosphatase (ACP) activity not only increased significantly (*p* < 0.05) in 0.2 mg/L treatment group, but also decreased significantly (*p* < 0.05) compared with the increase in metamifop concentration. However, in all treatment groups, alkaline phosphatase (AKP) activity was significantly lower than that in the control group (*p* < 0.05). In the inflammatory response, *TNF-α* and *IL-1β* expression levels in the NF-κB signaling pathway decreased significantly (*p* < 0.05) with the increase in metamifop concentration, while *IL-8* expression level in the same signaling pathway increased significantly (*p* < 0.05) in treatment groups. The expression levels of genes related to apoptosis showed that apoptosis was promoted after exposure to metamifop. The results of the present study show that metamifop induced oxidative damage via a high level of ROS production, and then inhibited or damaged the defense systems of *M. albus*.

## 1. Introduction

Rice is a staple grain. In 2021, the rice planting areas in China reached approximately 29.93 million km^2^ [1]. Weeds threaten rice growth, and competition in growth areas between weeds and rice reduces the emergence rate and yield of rice by 50% and 57%, respectively [2]. To promote rice yield, herbicides are extensively applied for weed management in paddy fields. Aryloxy phenoxy propionate (AOPP) is a series of frequently used herbicides. As a new AOPP herbicide, metamifop is widely used in paddy fields. Metamifop inhibits acetyl-CoA carboxylase (ACCase) activity in vivo, and disturbs lipid synthesis, resulting in weed elimination [3]. With the advancement in green agricultural development, various planting-breeding models based on paddy fields have been popularized, such as the rice−shrimp model, rice−carp model, and rice−eel model [4,5,6]. However, using herbicides in paddy fields threatens the health of aquatic animals in co-culture models. Given that the half-life of metamifop in paddy fields is 21.5–40.8 d, and the application of metamifop in China is 180–270 g. a.i./hm^2^, it is pertinent to explore the toxicity of metamifop to aquatic animals in co-culture models [7].

Previous studies have shown that steady-state reactive oxygen species (ROS) levels in vivo are usually disturbed by herbicides [8,9]. Appropriately increasing ROS levels promotes immunity, wound repair, and growth, while excessive free radicals damage cellular constituents via processes such as lipid peroxidation, protein carbonylation, and DNA mutation [8,10]. Moreover, a high level of ROS leads to apoptosis or even necrosis [11]. Apoptosis is a form of spontaneous programmed cell death regulated by genes to maintain homeostasis and better adapt to the living environment [12]. When organisms suffer from exogenous pollution, ROS are overproduced, and organisms may spontaneously form a detoxification mechanism called “oxidative stress” [11]. In the antioxidant system, antioxidant-related enzymes such as superoxide dismutase (SOD), glutathione peroxidase (GPX), and catalase (CAT) perform primary functions [13]. The immune response to oxidative stress is also important in aquatic animals [14,15]. Fish maintain a low level of acquired immunity, and innate immunity is their main defense system against pathogen infection [16]. The activation of immune-related enzymes is the first line of defense against exogenous stress in vivo. Acid phosphatase (ACP) and alkaline phosphatase (AKP) are important hydrolytic enzymes in fish [17,18]. These two enzymes are not only involved in the digestion, absorption, and transportation of nutrients, but also constitute an important detoxification system in fish that significantly affect their immune and antioxidant systems [19,20]. The liver is the main detoxification organ in fish and the main enrichment site of exogenous pollutants, but exogenous pollutants can inhibit the activity of liver metabolic enzymes [21]. Therefore, the activities of ACP and AKP in liver are more significantly affected by exogenous pollutants, and are important indexes to measure the immune response. Cellular immunity is an equally as essential part of the immune defense system as immune-related enzymes. Leukocytes (white blood cells, WBCs) are an important cellular immune factor, and lymphocytes, macrophages, and granulocytes are the main forms of WBCs. WBC synthesize antibacterial compounds and destroy pathogens [22]. The inflammatory response is an innate immune defense response of fish via oxidative stress, ultraviolet light, pathogen infection, and mechanical injury [23]. The inflammatory response is a beneficial process to contain and eliminate pathogens. However, an excessive inflammatory response can cause an inflammatory storm, inducing apoptosis, scorch death, necrosis, and other lesions [24].

*Monopterus albus* is an important commercial freshwater species in China and is popular because of its good texture and nutrition. As the main breeding object in the rice−eel model, *M. albus* is vulnerable to metamifop stress [25]. The effects of metamifop on the endocrine and ammonia metabolism in *M. albus* have been studied before [26,27]. However, the toxic mechanism of metamifop in *M. albus* is unclear. In this study, the potential toxicity and toxic mechanism of metamifop in *M. albus* were revealed by measuring relevant physiological and biochemical indexes, histological observation, and analyzing gene expression in *M. albus* after a 96 h exposure to metamifop.

## 2. Materials and Methods

### 2.1. Chemicals

Metamifop (C_23_H_18_CIFN_2_O_4_, CAS: 256412-89-2) was purchased from Hubei Jiangmin Taihua Chemical Co., Ltd. (Hubei, China). According to the method of Zhao et al. [28], a 1000 mg/L metamifop stock solution was prepared with de-chlorinated water, and 0.08% acetone and 0.0008% Tween-80 (*v*/*v*) was used for solubilization. Then, the metamifop stock solutions were diluted with processed de-chlorinated water (including 0.08% acetone and 0.0008% Tween-80 (*v*/*v*)).

### 2.2. Experimental Design

Healthy juvenile *M. albus* (12.60 ± 0.91 g in weight, 26.34 ± 0.78 cm in length) with strong vitality and no damage to the body surface were collected from Luoma Lake, Jiangsu Province, China. After the *M. albus* were transported to the laboratory, they were placed in a spare tank immediately to adapt to the water. The first 48 h were a settling-in period to minimize the stress caused by environmental changes. Then, during the acclimation period, the *M. albus* were placed in a polyethylene tank (5 m × 0.8 m × 0.4 m, water depth 0.3 m) at College of Modern Fisheries Industry (Huaiyin Normal University, Huai’an, China) for 1 week, where the pH, dissolved oxygen, water temperature, and photoperiod were 7.8 ± 0.1, 9.0 ± 0.5 mg/L, 20 ± 2 °C, and 14:10 (light:dark), respectively. Fish were fed with earthworms once a day at 7:30 p.m. In addition, the mortalities during the acclimation period were 2–5%. The conditions during the acclimation period were in line with the standards of OECD (2019) [29]. De-chlorinated water was used for the blank group, and the processed de-chlorinated water (containing 0.0008% Tween and 0.08% acetone) was used as the negative control. According to previous studies by our laboratory, the 96 h LC_50_ of metamifop to *M. albus* was 0.785 mg/L [26,27]. Combined with the application of metamifop in paddy fields [7], 0.2, 0.4, 0.6, and 0.8 mg/L metamifop were set as treatment groups. The 1000 mg/L metamifop stock solutions were diluted as 250, 500, 750, and 1000 mg/L to add into 12 L experimental water. A quantity of 9.6 mL diluted solution of each concentration was added into each treatment group to ensure the content of acetone and Tween-80 were equal to those in the negative control. Actual metamifop concentrations in water were determined by high performance liquid chromatography (HPLC). A total of 144 healthy *M. albus* were randomly divided into 6 groups (blank group, negative control, and four treatment groups) with 3 replicates. The *M. albus* were placed in culture tanks with volume specification of 40 cm × 30 cm × 15 cm and water depth of 10 cm. The tanks used in the experiment were covered with gauze to prevent the *M. albus* from escaping, and tiles were used as shelter. Physicochemical parameters of water were pH 7.8 ± 0.1, 9.0 ± 0.5 mg/L dissolved oxygen, 20 ± 2 °C temperature, and a 14:10 (light:dark) photoperiod. The experimental water was updated every 24 h, and the metamifop solution was re-added to maintain the metamifop concentration. No feeding was performed during the experiment.

### 2.3. Sample Collection

After a 96 h exposure to metamifop, the *M. albus* were anesthetized with MS-222 (Sigma-Aldrich Co., St. Louis, MO, USA). Blood samples were collected by severing the caudal peduncle to obtain the white blood cell count. Then, the *M. albus* were decapitated on ice, and liver tissues were collected, frozen in liquid nitrogen, and stored at −80 °C for further analyses of other biochemical indexes and histological observation.

### 2.4. White Blood Cell Count

A white blood cell dilution kit (Beijing Solarbio Science & Technology Co., Ltd., Beijing, China) was used for WBC counting, and the following steps were performed according to the instructions: 20 µL of blood was added into a 0.38 mL white blood cell dilution and mixed well. After all mature red blood cells were dissolved in the white blood cell dilution, 10 µL of the mixed solution was added to a hemocytometer, and white blood cells were counted under a microscope (XSP-10CA, Shanghai Youke Instrumentation Co., Ltd., Shanghai, China). Then, the number of white blood cells in the blood was calculated. Three replicates of each group were performed.

### 2.5. ROS Production Assay

Liver tissues were homogenized in 0.4 mL of Tris buffer (10 mmol/L Tris-HCl, 0.1 mmol/L EDTA-2Na, 10 mmol/L sucrose, and 0.8% NaCl, pH = 7.4) on ice and centrifuged at 12,000 rpm for 10 min at 4 °C to obtain the supernatant for testing. Dimethyl sulfoxide (DMSO, C_2_H_6_SO, CAS: 67-68-5, Beijing Solarbio Science & Technology Co., Ltd., Beijing, China) was dissolved in 20 µL of supernatant, 5 µL of 2′, 7′-Dichlorofluorescin diacetate (DCFH-DA, C_24_H_16_Cl_2_O_7_, CAS: 4091-99-0, Beijing Solarbio Science & Technology Co., Ltd., Beijing, China), and 100 µL of normal saline at a final concentration of 10 µmol/L, and this reaction system was incubated at 37 °C for 30 min under shade. Then, the optical density (OD) was determined with a microplate reader (Varioskan Lux, Thermo Scientific Co., Ltd., Singapore) at 485/530 nm (excitation wavelength/emission wavelength). The Tris buffer was determined as background fluorescence. ROS production was presented in florescence units [30]. Three replicates of each group were performed.

### 2.6. Related Enzyme Activity, MDA Content, and PCO Content Assay

ACP activity, AKP activity, SOD activity, GPX activity, CAT activity, MDA content, and PCO content in the liver were all determined following the instructions of the kits obtained from Beijing Solarbio Science & Technology Co., Ltd. (Beijing, China). Three replicates of each group were performed.

### 2.7. Histological Analysis

Liver tissues were fixed in a 4% paraformaldehyde solution for 24 h. The fixing solution was cleared with 70% ethanol. Then, the samples were dehydrated with 70% ethanol for 40 min, 80% ethanol for 40 min, 95% ethanol for 1 h, and 100% ethanol for 1 h. After dehydrating, the samples were soaked in a mixture of xylene and ethanol (1:1) for 30 min, and then in xylene for 1 h. The processed samples were embedded in paraffin at 55 °C, followed by sectioning and baking, and 5 µm paraffin sections were obtained. Then, a part of each section was stained with hematoxylin & eosin (H&E), sealed with natural gum, and finally observed under a light microscope (Leica DM2000 LED, Leica Instruments Co., Ltd., Wetzlar, Germany) and photographed with a 3DHISTECH scanner (Jinan Tangier Electronics Co., Ltd., Jinan, China).

### 2.8. Gene Expression

Total RNA was extracted from the liver samples with TRIzol reagent (CoWin Biotech Co., Ltd., Taizhou, China). Nano drop, Qubit 2.0, and Agilent 2100 were used to analyze the purity, concentration, and integrity of the RNA samples, respectively. Single-strand cDNA was synthesized using the HiFiScript cDNA Synthesis Kit (CoWin Biotech Co., Ltd., Taizhou, China) following the instructions. The LightCycler^®^ 480 II RT-qPCR instrument (Roche, Switzerland) was used to detect gene transcripts. Primer 6.0 software was used to design primers (Table 1). A quantity of 20 µL of reaction system included 10 µL of SYBR Premix Ex Taq (2×), 2 µL of each primer, 2 µL of cDNA, and 6 µL of ddH_2_O. Cycling was performed at 95 °C for 5 min, followed by 40 cycles at 95 °C for 10 s, 60 °C for 30 s, and 72 °C for 30 s. The 2^−ΔΔCt^ method was used to analyze the data. Three replicates of each group were performed.

### 2.9. Statistical Analysis

All data are presented as mean ± standard deviation (mean ± SD). Prior to data analysis, normality of the data was tested with the Shapiro−Wilk test, and homogeneity of variances was tested using Leven’s test. The data were analyzed using one-way ANOVA, followed by Duncan’s multiple comparison. *p* < 0.05 was considered statistically significant. All statistical analyses were performed using SPSS 26.0 software. All figures were drawn with GraphPad Prism 9.

## 3. Results

### 3.1. Solvent and Metamifop in Water

The statistical analysis showed that the solvent had no influence on the indexes in the present study. Thus, the negative control was set as the control group. The HPLC analysis of treatment groups showed that the actual metamifop concentrations ranged from 93% to 120% of all nominal concentrations (Table 2). Since the water in treatment groups was updated daily and metamifop concentrations were measured before and after the water update, the actual concentrations could be represented as nominal concentrations.

### 3.2. Effects of Metamifop on ROS Production, MDA Content, and PCO Content

**Figure 1:**
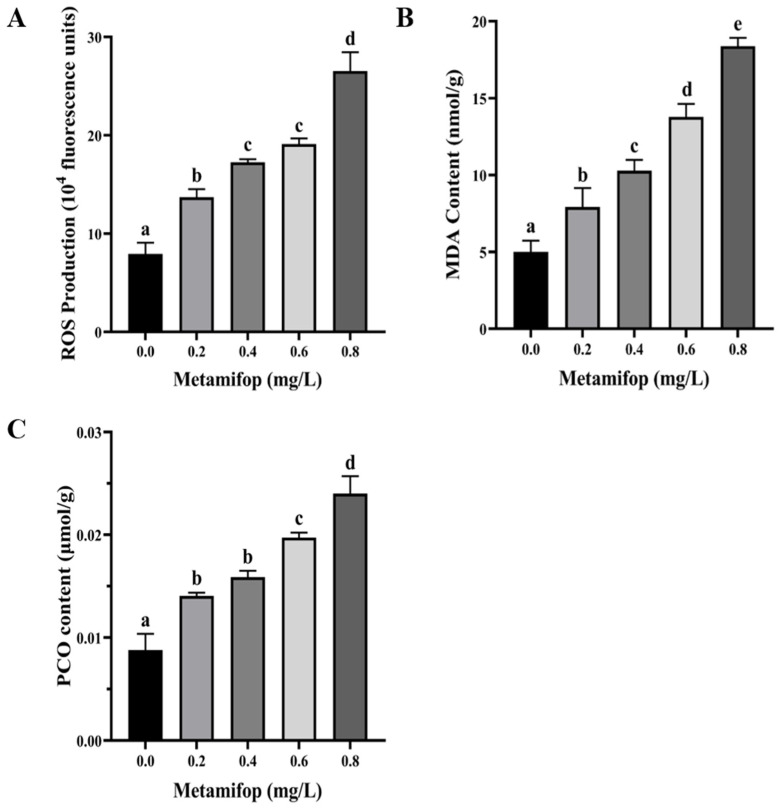
Effects of metamifop on ROS production, MDA content, and protein carbonyl in *Monopterus albus*. (**A**) ROS production; (**B**) MDA content; (**C**) PCO content. The data are presented as mean ± SD, three replicates of each group (n = 3). Different labels indicate significant differences between experimental groups (*p* < 0.05).

ROS production, MDA content, and PCO content all increased with the increasing metamifop concentration (Figure 1). In addition, the three indexes of the treatment groups were all significantly higher than those of the control group (*p* < 0.05).

### 3.3. Effects of Metamifop on Enzyme Activity

**Figure 2:**
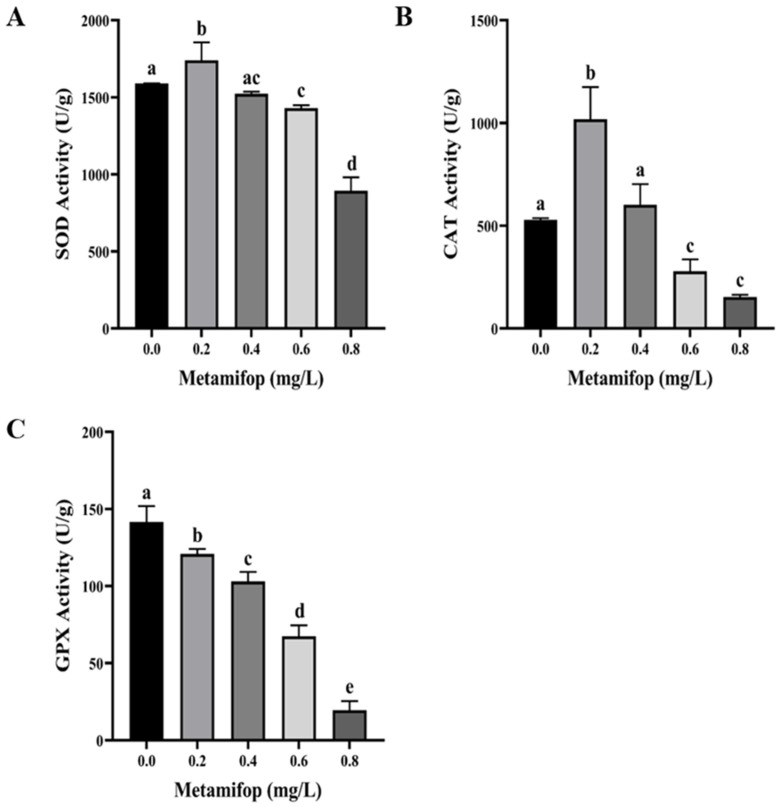
Effects of metamifop on antioxidation in *Monopterus albus*. (**A**) SOD activity; (**B**) CAT activity; (**C**) GPX activity. The data are presented as mean ± SD, three replicates of each group (n = 3). Different labels indicate significant differences between experimental groups (*p* < 0.05).

SOD and CAT activities first increased and then decreased with the increasing metamifop concentration (Figure 2A,B). In the 0.2 mg/L treatment group, SOD and CAT activities were significantly higher than those in the control group (*p* < 0.05). In 0.6 and 0.8 mg/L treatment groups, SOD and CAT activities were significantly lower than those in the control group (*p* < 0.05), while the two enzymes’ activities were lower in the 0.4 mg/L treatment group than in the control group, without a significant difference (*p* > 0.05). GPX activity decreased with the increasing metamifop concentration (Figure 2C). GPX activity in all treatment groups was significantly lower than that in the control group (*p* < 0.05).

**Figure 3:**
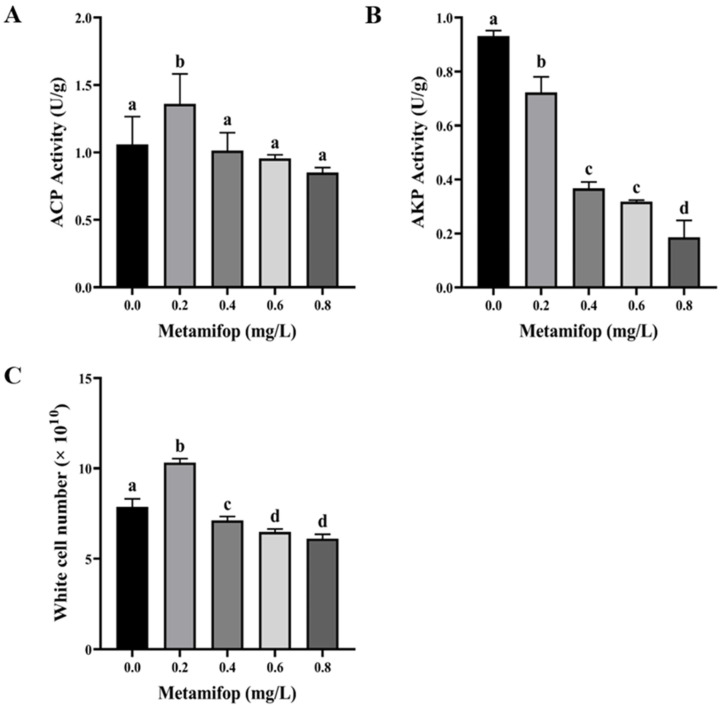
Effects of metamifop on immunity in *Monopterus albus*. (**A**) ACP activity; (**B**) AKP activity; (**C**) white cell number. The data are presented as mean ± SD, three replicates of each group (n = 3). Different labels indicated that there were significant differences between experimental groups (*p* < 0.05).

ACP activity first increased and then decreased with the increasing metamifop concentration (Figure 3A). ACP activity in the 0.2 mg/L treatment group was significantly higher than that in the control group (*p* < 0.05), and ACP activities in 0.4, 0.6, and 0.8 mg/L treatment groups were lower than those in the control group without significant differences (*p* > 0.05). AKP activity decreased with the increase in metamifop concentration (Figure 3B) and was significantly lower in all treatment groups than in the control group (*p* > 0.05).

### 3.4. Efftct of Metamifop on White Cell Number

As shown in Figure 3C, white cell number (WCN) increased as the metamifop concentration increased, where WCN first increased and then decreased. In the 0.2 mg/L treatment group, WCN was significantly higher than that in the control group (*p* < 0.05). In 0.4, 0.6, and 0.8 mg/L treatment groups, WCN was significantly lower than that in the control group (*p* < 0.05).

### 3.5. Effect of Metamifop on Histology

**Figure 4:**
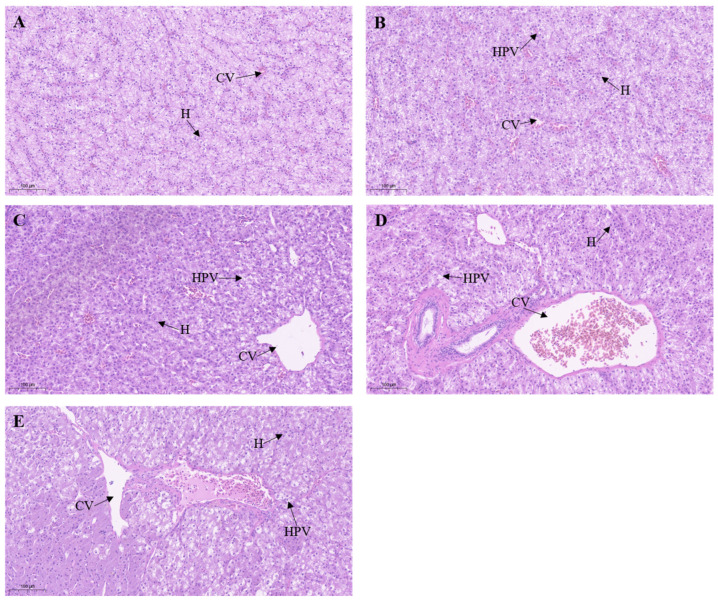
H&E-stained sections of liver tissues in *Monopterus albus* at different metamifop concentrations. (**A**) control group; (**B**) 0.2 mg/L treatment group; (**C**) 0.4 mg/L treatment group; (**D**) 0.6 mg/L treatment group; (**E**) 0.8 mg/L treatment group. H: Hepatocyte; HPV: Hepatocyte vacuolation; CV: Central venous. Scale bar = 100 μm.

After a 96 h exposure to metamifop, the histological observations of liver tissues are shown in Figure 4. Hepatocytes in the control group were arranged neatly around central venous, hepatocytes were structurally intact, and intercellular boundaries were clear. In the 0.2 mg/L treatment group, hepatocyte vacuolation increased and the central venous enlarged. With the increasing metamifop concentration, the central venous was distorted and enlarged, arrangement of hepatocytes was disordered, hepatocytes were swollen, hepatocyte vacuolation increased, and liver tissues were damaged.

### 3.6. Effects of Metamifop on Inflammatory-Related Genes

**Figure 5:**
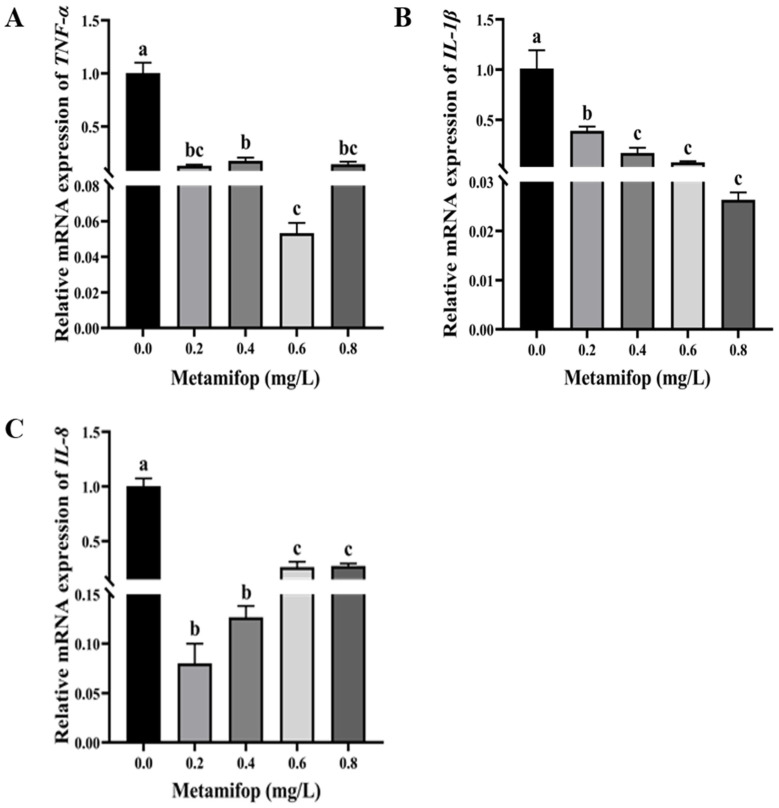
Effects of metamifop on expression level of inflammation-related genes in *Monopterus albus*. (**A**) expression of *TNF-α*; (**B**) expression of *IL-1β*; (**C**) expression of *IL-8*. The data are presented as mean ± SD, three replicates of each group (n = 3). Different labels indicated that there were significant differences between experimental groups (*p* < 0.05).

The effects of metamifop on the expression levels of inflammatory-related genes are shown in Figure 5. *TNF-α* expression levels in treatment groups were significantly lower than those in control group (*p* < 0.05). *IL-1β* expression levels decreased with the increasing metamifop concentration and decreased significantly compared with the control group (*p* < 0.05). *IL-8* expression level first decreased and then increased with the increasing metamifop concentration. *IL-8* expression levels in all treatment groups were significantly lower than those in the control group (*p* < 0.05).

### 3.7. Effects of Metamifop on Apoptosis-Related Genes

**Figure 6:**
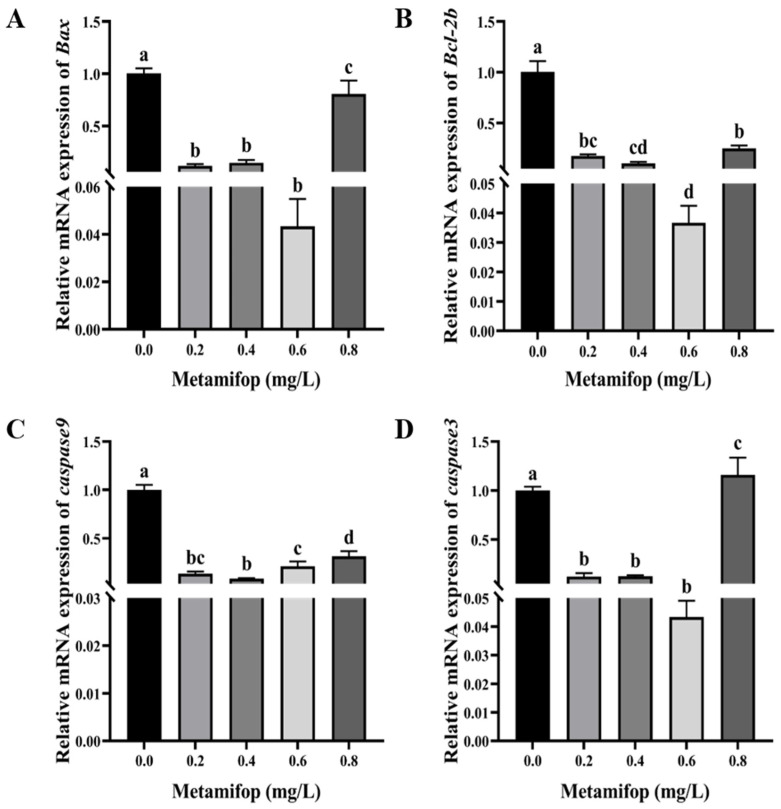
Effects of metamifop on expression level of apoptosis-related genes in *Monopterus albus*. (**A**) expression of *Bax*; (**B**) expression of *Bcl-2b*; (**C**) expression of *caspase-9*; (**D**) expression of *caspase-3*. The data are presented as mean ± SD, three replicates of each group (n = 3). Different labels indicated that there were significant differences between experimental groups (*p* < 0.05).

The effects of metamifop on the expression levels of apoptosis-related genes are shown in Figure 6. The expression levels of *Bax*, *Bcl-2b*, and *caspase9* first decreased and then increased with the increasing metamifop concentration, while the expression levels of the three genes in all treatment groups were significantly lower than that in the control group (*p* < 0.05). *caspase3* expression levels showed a similar trend with the increasing metamifop concentration. However, in the 0.8 mg/L treatment group, *caspase3* expression level was significantly higher than that in the control (*p* < 0.05), and in 0.2, 0.4, and 0.6 mg/L treatment groups, the expression levels were significantly lower than those in the control group (*p* < 0.05). In addition, although the expression levels of *Bcl-2b* and *Bax* decreased, the ratio of *Bax* to *Bcl-2b* increased with the increasing metamifop concentration. Moreover, the ratios in 0.4, 0.6, and 0.8 mg/L treatment groups were 1.45, 1.72, and 2.07, respectively, which were higher than that in control group.

## 4. Discussion

Herbicide abuse in recent decades has negatively affected non-target organisms, leading to suppression of defense responses or even damage to defense systems, including the antioxidant and immune systems, in aquatic animals [11,14]. Metamifop is a widely used herbicide in paddy fields, and its application has reached 180 g a.i./hm^2^ [7], which exceeds the exposure doses used in the present study. As an important commercial aquatic animal in the rice−fish co-culture model in China, it is necessary to explore the toxic mechanism of metamifop in *M. albus*.

Like other pollutants, herbicides induce ROS overproduction in vivo [11]. Low levels of ROS defend invasion by bacteria and viruses, but excessive ROS levels lead to oxidative stress or oxidative damage [31]. After a 96 h exposure to metamifop, ROS production increased with the increasing metamifop concentration, which suggests that metamifop induced ROS production. A previous study found that mesotrione induced excessive ROS production in *Cyprinus carpio*, resulting in oxidative stress and DNA damage [32]. Once organisms suffer from excessive ROS, the antioxidant system is activated to eliminate the ROS to maintain homeostasis. SOD, CAT, and GPX are important antioxidant enzymes. When the antioxidant system is activated, SOD first converts O_2_^−^ into H_2_O_2_ and O_2_, and then CAT and GPX convert H_2_O_2_ into harmless H_2_O and O_2_ [33,34]. In the present study, SOD and CAT activities were higher in the 0.2 mg/L treatment group than those in the control group, but SOD and CAT activities decreased with the increasing metamifop concentration. The results indicate that 0.2 mg/L of metamifop activated the antioxidant system of *M. albus* to alleviate antioxidant stress, but the ROS level increased with the increasing metamifop concentration, and the excessive ROS damaged the antioxidant system, leading to a decrease in the antioxidant capacity. This is similar to a study on cadmium in *Macrobrachium nipponense*, wherein after a 96 h exposure to cadmium, low concentrations of cadmium (0.01 and 0.02 mg/L) activated the antioxidant defense system, and a high concentration (0.04 mg/L) inhibited the antioxidant defense system [35]. Although GPX activity did not show a similar trend to that of the SOD and CAT activities, it demonstrated that the antioxidant capacity decreased with the increasing metamifop concentration. Excessive ROS damage cellular constituents, and as a final product of lipid peroxidation, MDA is important for measuring lipid peroxidation and oxidative damage [36]. In the present study, MDA content increased with the increasing metamifop concentration. A similar outcome was found in a study of pendimethalin on *Oreochromis niloticus* [34]. The increase in MDA content suggests that the antioxidant defense system failed to prevent the overproduction of free radicals, and the amino acid side chain then underwent irreversible covalent modification to form carbonyl groups, causing changes in protein structure and function [37,38]. As another important biomarker of oxidative stress, PCO content indicates the degree of oxidative stress [39]. In the present study, PCO content increased with the increasing metamifop concentration. The results show that ROS production increased with the increasing metamifop concentration, whereby the antioxidant system first participated in the defense against excessive free radicals. However, when ROS accumulated to such an extent that they could not be eliminated, free radicals damaged cellular constituents, inducing lipid peroxidation and protein carbonylation, resulting in oxidative damage.

The immune system is another essential defense system in fish that is as important as the antioxidant system. In the innate immune system, ACP and AKP are important hydrolases that promote disease and stress resistance abilities [40] and whose activities indirectly reflect tissue damage. Thus, ACP and AKP activities are commonly used as indicators of fish health status. Besides humoral immunity, cellular immunity is another important part of the innate immune system. Fluctuations in blood parameters are typical of fish exposed to herbicides [41]. WBCs are a type of immune and self-protective cell that engulfs and degrades pathogens and tissue debris in fish. When exogenous substances invade organisms, the number of WBCs significantly increases [22]. In the present study, ACP activity increased in the 0.2 mg/L treatment group, while it decreased with the increasing metamifop concentration. Moreover, AKP activity decreased with the increasing metamifop concentration. A previous study showed that dimethoate inhibited ACP and AKP activities in *Danio rerio* [42]. In the present study, WCN increased in the 0.2 mg/L treatment group, and then decreased with the increasing metamifop concentration. Previous studies showed that WCN in *Cyprinus carpio* L. decreased upon exposure to metribuzin, and glyphosate also decreased WCN [43,44]. The results of the present study show that *M. albus* in 0.2 mg/L treatment group tried to activate the immune system to cope with oxidative stress. However, oxidative stress intensified with the increasing metamifop concentration, causing oxidative damage, and probably leading to immune system disruption.

The inflammatory response is a direct link between the immune system and injury. In the present study, the expression levels of three pro-inflammatory factor genes (*TNF-α*, *IL-1β*, and *IL-8*) were used to measure the immune response. *TNF-α*, *IL-1β*, and *IL-8* bind to receptors, triggering the intracellular NF-κB signaling pathway, which triggers the immune response [19]. Fish tumor necrosis factor (TNF) ligands are produced by macrophages, and TNF ligands are important for regulating immune function, metabolism, and morphological development. The main functions of *TNF-α* in fish involve inflammation, apoptosis, fat metabolism, and organ regeneration [45,46,47,48,49]. In the present study, *TNF-α* expression levels were inhibited after exposure to metamifop. A previous study showed that a high dose of *Nocardia* or chronic infection decreased *TNF-α* expression level in *Paralichthys olivaceus*, indicating that the *TNF-α* expression level was related to the concentration of influencing factors and exposure time [50]. *IL-1β* and *IL-8* are essential in the initiation and maintenance of inflammation [51]. When organisms suffer from the stress of environmental factors, *IL-1β* participates in activating the inflammatory response and resisting bacterial infection [52]. *IL-8* maintains the inflammatory response to achieve sterilization and then repairs infected tissues [53]. In the present study, metamifop decreased the expression levels of *IL-1β* and *IL-8*. These results are similar to the effects of glyphosate exposure on gills and intestines of *European seabass* and the effects of high concentrations of propiconazole on *Danio rerio* embryos [54,55]. The results of the present study indicate that metamifop had a strong negative effect on the expression levels of pro-inflammatory genes in *M. albus*, and the immune capacity of *M. albus* decreased with the increasing metamifop concentration. However, *IL-8* expression level increased with the increasing metamifop concentration, indicating that the inflammatory response was actively mobilized to sterilize and repair infected tissue. This further proved that metamifop caused oxidative damage to the liver tissues of *M. albus*, resulting in infection. Therefore, in the case of impaired humoral and cellular immunity, *M. albus* initiated the inflammatory response to cope with stress. Moreover, through histological observation, it can be found that, with the increasing metamifop concentration, liver tissues were seriously damaged and vacuolated, which also led to disruption of immunity and antioxidation.

Oxidative stress or oxidative damage further induces cell apoptosis. *Bcl-2*, *Bax* and the *caspase* family are key genes directly involved in the regulation of apoptosis [56,57]. *Bax* activates channels of apoptosis-inducing factors in the inner mitochondrial membrane, so apoptosis-inducting factors such as cytochrome C (Cyt C) enter the cytoplasm and activate caspase 3 to induce apoptosis [58]. On the contrary, the main function of *Bcl-2* is to inhibit apoptosis. *Bcl-2* not only inhibits the formation of apoptosis-inducing factors, but also fixes the leaked apoptotic premise on the mitochondrial membrane to inactivate it [59]. When apoptosis occurs, the ratio of *Bax* to *Bcl-2* increases, resulting in the release of Cyt C from mitochondria. This activates the release of caspase-3 and caspase-9 and then induces apoptosis [60]. In the present study, expression levels of *Bax* and *Bcl-2b* were inhibited by metamifop, but the ratio of *Bax* to *Bcl-2b* increased. At the same time, expression levels of *caspase9* and *caspase3* showed a decreasing and then increasing trend with the increasing metamifop concentration. The results show that expression levels of *caspase9* and *caspase3* decreased in 0.2 and 0.4 mg/L treatment groups, and the apoptotic pathway was not actively involved in the defense response, or it was not initiated. However, more ROS accumulated in vivo with the increasing metamifop concentration, causing lipid peroxidation and protein carbonylation, leading to oxidative damage. The antioxidant and immune systems were unable to cope with the stress, and apoptosis was initiated to undergo the defense response.

## 5. Conclusions

Metamifop induced a high level of ROS production, which damaged cellular constituents and liver tissues, resulting in lipid peroxidation and protein carbonylation. Oxidative stress was intensified to oxidative damage with the increasing metamifop concentration, and the activities of the antioxidant and immune systems were then inhibited or even disrupted. Meanwhile, apoptosis was activated in response to stress induced by metamifop. In conclusion, metamifop induced oxidative damage and then impaired the defense systems of *M. albus*.

## Figures and Tables

**Table 1:** The primers for qRT-PCR in *Monopterus albus*.

Genes	Primer Sequence (5′–3′)
*Bax*	F: GGAGCAAGGTGGCTGGGTAA
	R: GTGGACTCCCAATCCTTAGACA
*Bcl-2b*	F: AGCCCACAAAACCACCACA
	R: GACCACACAACCACCATCTCA
*caspase-9*	F: ATGTTGATGATGGTTGGTGCC
	R: CTTTGCGTGGGTGATGCTT
*caspase-3*	F: GGTTCTGACCCTTACCGCTAC
	R: TGTCCCATCTGCTAACGTGGA
*TNF-α*	F:CCTTAGCCACACAGTGATGCG
	R:CCCAGGCTCATCTTCCAGGT
*IL-1β*	F:ACCTCATTATCGCCACGGAG
	R:ATTTTACGGTTGTCGCTGCC
*IL-8*	F:TACTGGTTCTGCTTACTGTCGC
	R:CAAATCTTTTGCCCATCCCT
*β-actin*	F:TCAACACGCCTGCCATGTAT
	R:CGCTCAGCTGTGGTAGTGAA

**Table 2:** Actual metamifop concentration in experimental water over 24 h.

Actual Concentration (mg/L)	Nominal Metamifop Concentration (mg/L)			
	0.2	0.4	0.6	0.8
0 h	0.24 ± 0.02	0.43 ± 0.04	0.66 ± 0.02	0.85 ± 0.01
24 h	0.19 ± 0.01	0.38 ± 0.04	0.56 ± 0.05	0.76 ± 0.03

The data are presented as mean ± SD, three replicates of each group (n = 3).

## Data Availability

Data will be made available on request.
